# Cutaneous Metastasis From Rectal Adenocarcinoma Mimicking Vegetative Genital Herpes: A Case Report

**DOI:** 10.7759/cureus.103666

**Published:** 2026-02-15

**Authors:** Alejandra González Uribe, Verónica L González Sánchez, Diana S Blanquet Campos, Martha A Aceves Villalvazo

**Affiliations:** 1 Department of Internal Medicine, Hospital Regional “Dr. Valentín Gómez Farías” ISSSTE, Guadalajara, MEX; 2 Department of Dermatology, Hospital Regional “Dr. Valentín Gómez Farías” ISSSTE, Guadalajara, MEX

**Keywords:** atypical herpetic infection, cutaneous metastasis, genital herpes, kras mutation, rectal adenocarcinoma

## Abstract

Colorectal cancer is the third most common cancer worldwide. Cutaneous metastases are rare and usually represent advanced disease. and their diverse clinical manifestations may mimic inflammatory or infectious dermatoses, leading to delayed diagnosis and poor outcomes.

We present a case of a 70-year-old woman with a history of moderately differentiated rectal adenocarcinoma treated with neoadjuvant radiotherapy and surgery who developed metastatic disease with a KRAS G12V mutation. During follow-up, she presented with vulvar, perineal, inguinal, and gluteal erythematous papules, nodules, and simulating vesicles, initially misdiagnosed as an atypical herpetic infection. Histopathological examination of punch biopsies revealed gland-forming malignant epithelial cells consistent with cutaneous metastasis from colorectal adenocarcinoma.

Cutaneous metastases from colorectal cancer show heterogeneous and sometimes rare presentations, including herpetiform and pseudotumoral patterns. Vulvar involvement is exceptionally uncommon therefore prompt recognition and histopathological confirmation are essential in patients with persistent or atypical lesions to avoid misdiagnosis and allow timely management.

## Introduction

Colorectal cancer ranks third worldwide in both incidence and mortality, being the most frequent type of cancer in men and the second most common in women. Metastatic disease occurs with an overall frequency of 20%, with the most common sites of metastasis being the liver (70%), lung (32%), peritoneum (21%), and skin [1-3].

Cutaneous metastases are uncommon (<5%), with few cases documented in the literature [4-6]. They typically appear within the first two years after identification of the primary tumor and/or surgical resection and may occur simultaneously in multiple organs. In approximately 20% of patients, they occur simultaneously with the diagnosis of the primary tumor and are found in 0.7-5% of patients with visceral neoplasms and in 10% of patients with metastatic cancer [1-5].

The most common route of dissemination is lymphatic; however, direct tumor implantation, intraluminal spread, and hematogenous dissemination have also been described [1]. It has been reported that in 50% of cases, the most frequent site of cutaneous metastasis from colorectal adenocarcinoma is the surgical scar from abdominal resection, likely related to proximity to the primary tumor or direct implantation of tumor cells during surgery. There is a theory regarding the Koebner phenomenon, characterized by a predilection for tumor cell implantation in sites of prior infection, trauma, or surgical intervention [1]. This is followed in frequency by skin adjacent to the primary tumor (30%), genital and perineal regions (15%), and extremities (11%) [1,3-6]. In approximately 70% of patients, cutaneous metastases present as a single lesion, whereas 30% develop multiple cutaneous lesions [3].

Multiple clinical presentations of cutaneous metastases have been described, including ulcers, papules, nodules, plaques, and dermal or subcutaneous nodules with or without pain, characterized by rapid growth on intact skin or mimicking inflammatory dermatoses [3,7], and on rare occasions, as a vesicular-maculo-papular eruption simulating infectious etiologies [1,8].

## Case presentation

A 70-year-old female patient with a prior diagnosis of moderately differentiated rectal adenocarcinoma in 2022 is presented, with no additional significant past medical history. She received neoadjuvant radiotherapy followed by colectomy with hysterosalpingo-oophorectomy. The patient remained in remission for 12 months. In September 2024, a cervical lesion secondary to metastatic rectal carcinoma with a KRAS mutation (G12V) was identified. First-line chemotherapy was initiated with the CAPOX regimen (capecitabine plus oxaliplatin), followed by maintenance therapy with bevacizumab.

In January 2025, the patient developed localized edema and erythema of the vulva and labia majora, accompanied by papules and simulating vesicles. These were initially managed by the surgical oncology service with acyclovir due to suspicion of atypical herpetic infection; however, no clinical improvement was observed, for which she was referred to dermatology.

**Figure 1:**
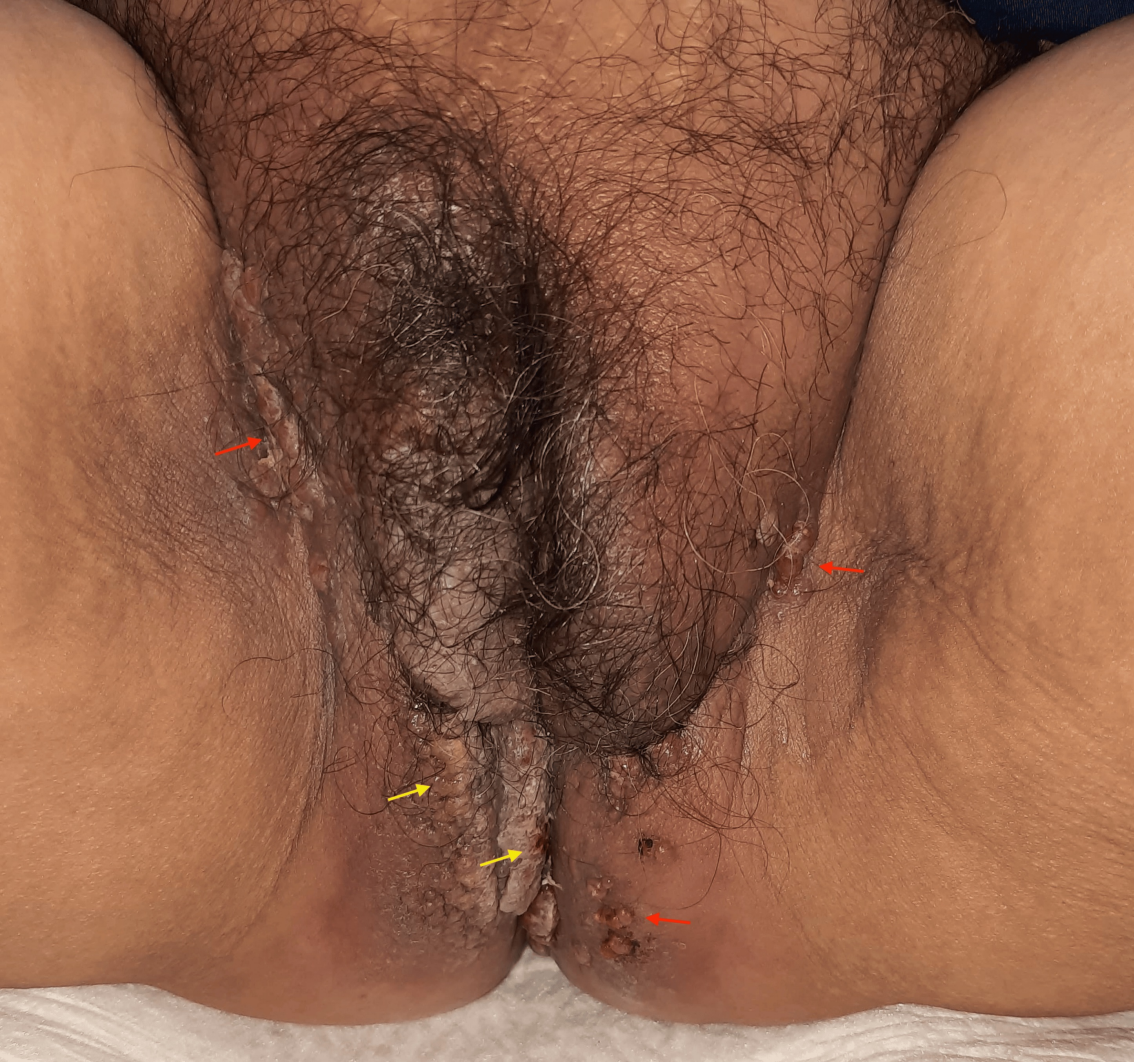
Initial impression of the dermatosis on physical examination Extensive erythematous to pink papules and nodules, coalescing into indurated plaques with poorly defined borders (red arrows); some lesions showed a vegetative and ulcerated surface with intergluteal extension (yellow arrows).

Dermatologic examination revealed a dermatosis involving the labia majora, inguinal folds, vulva, perineum, and right gluteal region, with erythematous and pink nodules measuring 3-8 mm with a shiny surface, isolated and confluent, forming indurated erythematous-pigmented plaques with poorly defined borders. Some lesions displayed a vegetative appearance, ulcerated on the right gluteal region. Intergluteal region showed extension to the inferior gluteal areas, labia majora, pubis, and inguinal folds, consisting of an indurated erythematous plaque with poorly defined borders causing retraction of subcutaneous tissue, along with numerous red to pink papular neoformations with shiny or eroded surfaces, measuring 3-8 mm, with a tendency to coalesce in the inguinal and intergluteal folds with a vegetative appearance (Figure 1).

**Figure 2:**
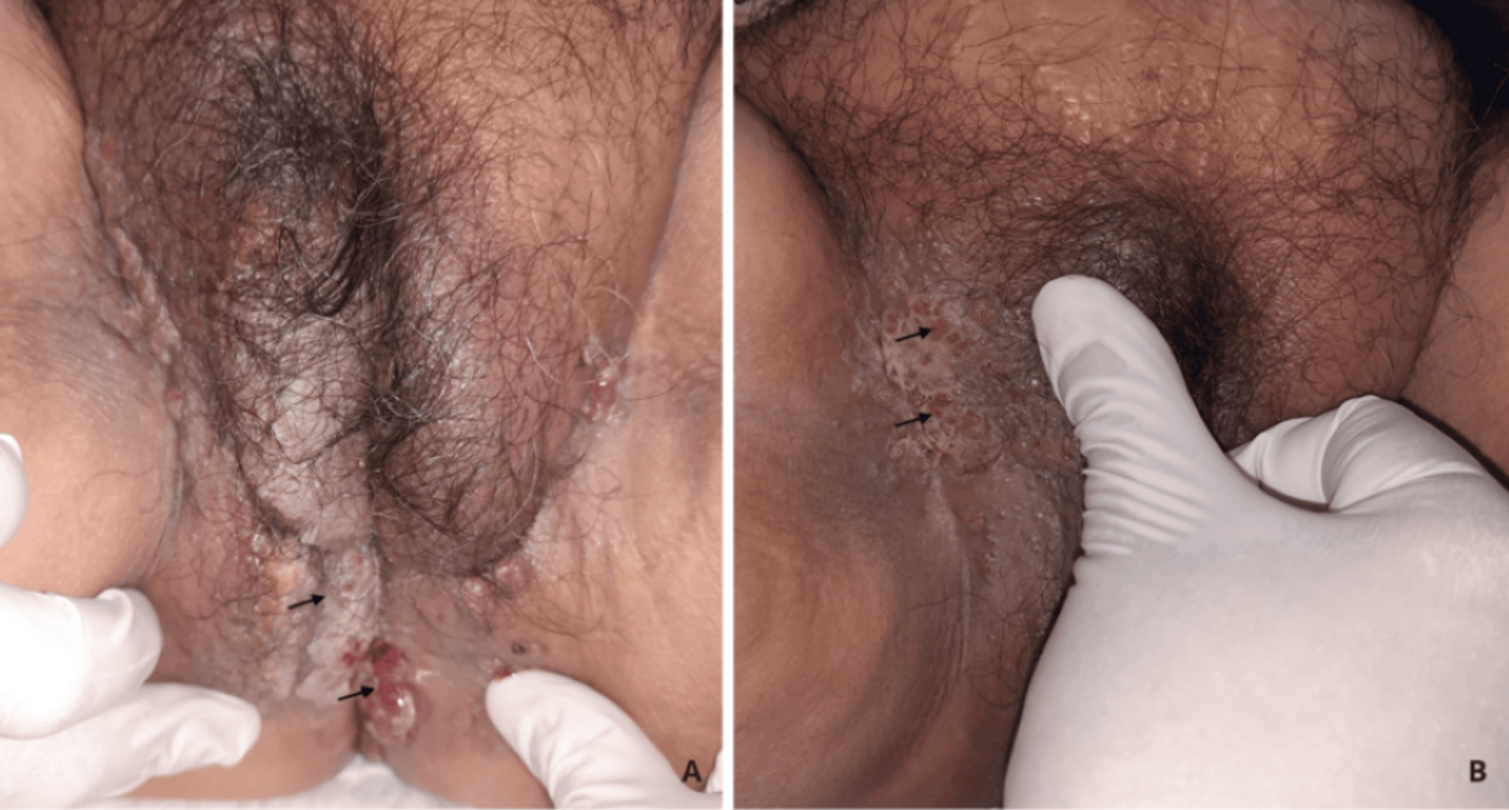
A: lesions displayed a vegetative appearance with ulcerated surface. B: Multiple pink and erythematous nodules.

On the right labium majus, perineum, and right gluteal region, a grayish verrucous-appearing plaque was observed, with areas of maceration and superficial ulceration (Figure 2).

**Figure 3:**
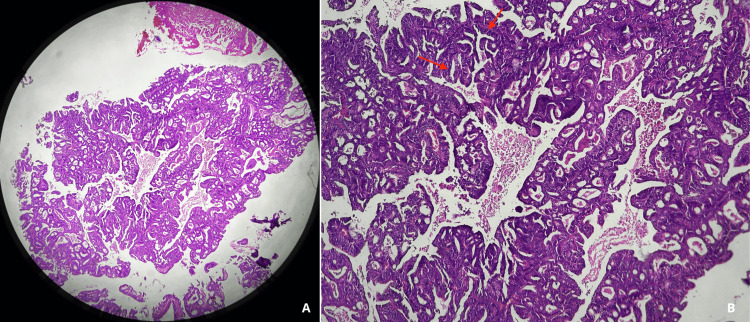
Histopathological findings (A) Low-power photomicrograph (H&E) showing a malignant tumor. (B) Higher-magnification view demonstrating neoplastic epithelial cells arranged in tubular and glandular structures (Red arrows).

Two punch biopsies were performed due to suspicion of other pseudotumoral infectious or neoplastic etiologies. Histopathological examination revealed a malignant tumor composed of neoplastic epithelial cells forming tubular and glandular structures, with pleomorphic nuclei, prominent nucleoli, and numerous atypical mitoses (Figure 3).

KRAS mutation testing was positive, consistent with cutaneous metastasis from colorectal carcinoma. Palliative management with second-line chemotherapy was initiated in March 2025, based on the FOLFIRI regimen (5-fluorouracil, irinotecan, and folinic acid).

## Discussion

Cutaneous metastases most commonly originate from well-differentiated mucin-secreting adenocarcinomas, and particularly in colorectal adenocarcinoma, cutaneous involvement is often complex and atypical in presentation [4,5].

Two clinical forms of presentation of cutaneous metastases from colorectal cancer have been described: the first with multiple visceral and cutaneous metastases at the time of presentation, and the second with the development of cutaneous metastases during follow-up or after resection of the primary tumor [3].

Several clinical variants of presentation have been reported, including erysipeloid, telangiectatic, neoplastic alopecia, erythema annulare centrifugum-like, and herpetiform patterns, the latter being particularly rare with few cases described in the literature, characterized by papulovesicles, nodules, or vesiculobullous lesions. Histologically, these metastases may present as adenocarcinoma, squamous cell carcinoma, or poorly differentiated carcinoma, typically demonstrating nodular configurations within the dermis with subsequent extension to the epidermis and subcutaneous tissue [4,7]. Colorectal cancer, known for its heterogeneous presentation, may give rise to hemophagocytic lymphohistiocytosis as a secondary immune dysregulation triggered by advanced malignancy, infection, or systemic anticancer therapies [8].

Vulvar metastases originating from colorectal cancer are exceptionally rare, with very few cases reported in the medical literature. Clinical presentations include bilateral pink papules on the labia majora, as well as friable subcentimeter tumors located in the anterior right introitus. Other reported manifestations include pain, vulvar discomfort, ulcerations, masses, and inguinal lymphadenopathy [9]. In the present case, vulvar dissemination is hypothesized to result from retroperitoneal lymphatic congestion and retrograde lymphatic spread.

Classic herpes simplex virus (HSV) infection is the leading cause of genital ulcer disease worldwide, especially HSV-2, and typically presents with papulo-vesicles eruptions or clusters of erosions [10,11]. However, in immunocompromised patients, less common clinical variants have been described, including nodules, hypertrophic vegetative plaques, ulcers, and pseudotumoral plaques [10]. Both primary and metastatic cutaneous malignancies have been described as mimicking HSV infections [11,12]. 

A case was reported of a patient who presented with ulcerated nodules in the genital area, demonstrating progressive enlargement. Physical examination revealed hypertrophic vegetative nodular lesions with ulcerated surfaces at the margins. A neoplastic etiology was initially suspected; however, histopathological examination revealed multinucleated cells with steel-gray, molded nuclei, findings consistent with HSV infection [10]. In another case, the patient initially reported red itching “herpes” on the vulva, followed by small solid papules and nodules that progressively enlarged and disseminated; some were smooth and shiny surface with vesicular changes. Histopathological examination was consistent with cutaneous metastasis of her previous malignancy [13]. 

This case series contributes to the literature by demonstrating that genital HSV lesions may manifest a tumoral morphology, manifesting as nodular herpetic lesions with atypical clinical features [10].

The American Society of Clinical Oncology (ASCO) [14] endorses physical examination with intentional assessment for cutaneous metastases in patients with confirmed colorectal adenocarcinoma. In patients with a history of malignancy who develop dermatoses unresponsive to targeted therapy after two weeks, cutaneous biopsy should be strongly considered [1]. Advanced diagnostic techniques, particularly immunohistochemistry, play a crucial role in confirming the diagnosis, typically demonstrating positivity for CDX-2 and CK20 in tumor cells [5,14].

Cutaneous metastases are more frequently observed in clinical stages II-IV, in a synchronous (52%) or metachronous (48%) manner, with presentation as an initial manifestation being exceptional (<1%). Prognosis in stage IV disease is poor, with a five-year survival rate below 5%, 50% survival at six months, and less than 40% at one year. However, a slightly more favorable outcome has been observed in patients younger than 55 years [3,15-17].

Cutaneous metastases from colorectal cancer generally represent a late event in advanced visceral malignancy and are associated with poor prognosis. Early recognition is critical, as timely intervention may prolong survival, since the average survival duration after identification of cutaneous metastases ranges from one to 34 months, with a mean survival of approximately 18 months [1,3,5-7].

## Conclusions

Cutaneous metastasis of colorectal cancer is an infrequent entity, with few cases reported in the literature; therefore, it is not usually considered the first clinical suspicion, in addition to the wide spectrum of clinical presentations, making initial misdiagnosis common.

Therefore, in the presence of atypical or persistent nodular lesions in patients with a history of malignant neoplasms, especially in those with atypical vesicular lesions that may mimic a herpetiform pattern, it must be considered as part of the differential diagnosis, as it may indicate disease progression or recurrence and poor prognosis.
